# White Matter Tract Alterations in Drug-Naïve Parkinson's Disease Patients With Excessive Daytime Sleepiness

**DOI:** 10.3389/fneur.2019.00378

**Published:** 2019-04-16

**Authors:** Amir Ashraf-Ganjouei, Ghazaleh Kheiri, Maryam Masoudi, Bahram Mohajer, Mahtab Mojtahed Zadeh, Pejman Saberi, Mehdi Shirin Shandiz, Mohammad Hadi Aarabi

**Affiliations:** ^1^Students' Scientific Research Center, Faculty of Medicine, Tehran University of Medical Sciences, Tehran, Iran; ^2^Non-Communicable Diseases Research Center, Endocrinology and Metabolism Population Sciences Institute, Tehran University of Medical Sciences, Tehran, Iran; ^3^Department of Radiology, Faculty of Medicine, Tehran University of Medical Sciences, Tehran, Iran; ^4^Department of Medical Physics, Zahedan University of Medical Sciences, Zahedan, Iran

**Keywords:** excessive daytime sleepiness, Parkinson's disease, diffusion MRI, connectometry, PPMI

## Abstract

Excessive daytime sleepiness (EDS) is relatively frequent in patients with Parkinson's disease (PD), having a prominent burden on patients' quality of life and causing dangerous events such as motor-vehicle accidents. Previous studies have indicated the role of certain neural tracts in the pathophysiology of sleep disturbances, especially in PD patients. We hypothesized that white matter integrity and connectivity might be altered in patients with PD and EDS. Therefore, this study investigated brain white matter microstructure alterations in patients with Parkinson's disease with EDS (PD-EDS) compared to healthy controls and PD patients without EDS (PD-nEDS). Diffusion MRI connectometry was used to carry out group analysis between PD patients with and without EDS and healthy individuals. EDS in PD patients is associated with decreased connectivity in the left and right fornix, left and right inferior longitudinal fasciculus (ILF), left inferior and middle cerebellar peduncles in comparison to PD-nEDS group. These differences between PD-EDS and PD-nEDS patients reflects microstructural changes with respect to sleep-related circuits, which can pave the way for future investigations considering EDS pathogenesis in Parkinson's disease.

## Introduction

Non-motor symptoms in Parkinson's disease (PD) have been given more attention during recent years, mainly due to their massive burden on patients' quality of life (1, 2). Affecting up to 60% of PD patients, excessive daytime sleepiness (EDS) is characterized by inappropriate sleepiness during the waking time. It could be present anytime in the course of PD, even before the appearance of the motor symptoms (3–5) and be regarded as a risk factor for the development of neurodegenerative diseases in future (6). Moreover, a longitudinal study revealed that the presentation of EDS progresses throughout the course of the disease from 4 to 41% in 8 years (7). Multiple factors are supposed to be associated with the prevalence of EDS including age, sex, presence of rapid eye movement (REM) sleep behavior disorder (RBD), mood disorders and cognitive impairment (5, 8–10). However, the underlying pathophysiology of EDS in PD is not understood completely. Based on the animal studies, there is growing evidence noticing dopamine as a significant contributor in the wake and sleep cycles (11). Moreover, since sudden sleep periods might lead to tragic events such as motor-vehicle accidents, elucidating the neuropathological mechanisms of EDS would be of great value, especially in PD patients.

Several studies have investigated cerebral structural changes in PD with EDS (PD-EDS) patients, using different imaging modalities. Single photon emission computed tomography (SPECT) imaging showed significant hypoperfusion in left temporal and parietal cortices in patients with EDS (PD-EDS) compared to patients without EDS (PD-nEDS) (7). Another SPECT study showed more severe nigrostriatal dysfunction in PD-EDS in comparison to PD-nEDS group (8). Yousaf et al. used Dopamine transporter SPECT imaging and revealed that increased DAT binding ratio in thalamus, proposing probable dopamine transporter upregulation in response to dopamine deprivation in PD-EDS patients compared to PD-nEDS patients (10). In another study, Yousaf et al. found the loss of dopaminergic activity in caudate nucleus as an indicator of EDS severity along with being a predictor of developing EDS over a period of 3 years (11).

Considering gray matter (GM), studies have shown that EDS is associated with regional brain atrophy in the middle medial cerebellar peduncle (12) as well as frontal, temporal, occipital, and limbic lobes in PD patients (13). However, a multimodal imaging study showed increased volume in the gray matter of the bilateral hippocampus and parahippocampal gyri. Furthermore, using diffusion tensor imaging (DTI), increased axial diffusivity (AD) was observed in certain white matter tracts including the left anterior thalamic radiation, corticospinal tract and superior longitudinal fasciculus (SLF) in PD-EDS patients (14). Another DTI study indicated that patients with EDS have reduced connectivity only in the fornix (15). Recently, resting-state functional MRI (rs-fMRI) revealed decreased regional homogeneity (ReHo) in the inferior frontal gyrus and left cerebellum, besides decreased functional connectivity (FC) of these regions in frontal and temporal lobes and cerebellum in PD patients suffering from EDS compared to PD-nEDS group (16). Moreover, in another rs-fMRI study, it was indicated that reduced functional connectivity is observed in the thalamostriatal network among PD-EDS patients (17). Although, all of the studies above provide evidence regarding brain alternations in PD-EDS patients, however, further studies are required to reveal new aspects of EDS development in PD patients.

DTI has been used widely by neuroscientists in the past decade for evaluating the white matter microstructure, especially in neurodegenerative disorders such as PD (18). However, due to certain limitations of end-to-end fiber tracking, connectometry was introduced as a novel approach to investigate white matter changes, using the concept of local connectome (19). Therefore, study variables can be tested for possible associations with local connectomes, which is defined by the degree of connectivity between adjacent voxels within a fascicle. In addition, Connectometry relies on the Spin Distribution Function (SDF) that measures the density of water diffusion in any direction, then reporting the peak SDF for each direction as quantitative anisotropy (QA). Consequently, we hypothesize structural networks may be responsible for exhibiting Parkinson related EDS and could be revealed using connectometry. This study aimed to investigate white matter microstructure alterations by using connectometry in patients with PD-EDS, in comparison to PD-nEDS and healthy individuals.

## Materials and Methods

### Participants

Participants involved in this research were recruited from Parkinson's Progression Markers Initiative (PPMI, http://www.ppmi-info.org/). The study was approved by the institutional review board of all participating sites in Europe, including Attikon University Hospital (Greece), Hospital Clinic de Barcelona and Hospital Universitario Donostia (Spain), Innsbruck University (Austria), Paracelsus-Elena Clinic Kassel/University of Marburg (Germany), Imperial College London (UK), Pitié-Salpêtrière Hospital (France), University of Salerno (Italy), and in the USA, including Emory University, Johns Hopkins University, University of Alabama at Birmingham, PD and Movement Disorders Center of Boca Raton, Boston University, Northwestern University, University of Cincinnati, Cleveland Clinic Foundation, Baylor College of Medicine, Institute for Neurodegenerative Disorders, Columbia University Medical Center, Beth Israel Medical Center, University of Pennsylvania, Oregon Health & Science University, University of Rochester, University of California at San Diego, University of California, San Francisco. Written informed consent was obtained from all participants before study enrolment. To be enrolled into the PPMI study, all patients were required to fulfill the following criteria: (1) met the standard diagnostic criteria for PD, (2) diagnosed within 2 years before the initial visit, (3) Hoehn & Yahr (H&Y) stage ≤ 2 at baseline, (4) demonstrated deficits on DaTscan imaging, and (5) not on any PD medication. Diffusion MRI images were obtained for 16 with EDS (9 females and 7 males), as indicated by the Epworth Sleepiness Scale scores > = 10, 45 PD patients without EDS (29 females and 16 males), and 17 healthy controls (10 females and 7 males). Table 1 shows the demographic and baseline level characteristics of participants in different groups.

**Table 1:** Demographic and baseline clinical information of patients with Parkinson disease.

**Feature**	**PD-EDS** **(*n* = 16)**	**PD-nEDS** **(*n* = 45)**	**HC** **(*n* = 17)**	*P*-value*
Age at diagnosis in years (mean ± SD)	62.31 ± 8.8	58.44 ± 9.3	61.36 ± 11.25	0.308
Male/Female no.	7/9	16/29	7/10	0.449
Handedness (L/R)	1/15	6/39	3/14	0.766
Education years (mean ± SD)	16.18 ± 2.4	14.6 ± 3.03	16 ± 2.89	0.086
Duration of disease in months (mean ± SD)	8.2 ± 7.03	7.92 ± 8.29	–	0.937
ESS (mean ± SD)	11.5 ± 1.75	5.62 ± 2.34	4 ± 2.54	0.000
University of Pennsylvania Smell Identification Test (UPSIT) (mean ± SD)	20.18 ± 7.75	23.37 ± 8.29	32.7 ± 5.16	0.000
H & Y stage (mean ± SD)	1.81 ± 0.4	1.71 ± 0.45	–	0.523
UPDRS III (mean ± SD)	28.18 ± 8.32	24.31 ± 6.88	–	0.072
GDS score (mean ± SD)	3.8 ± 1.72	4.2 ± 1.17	4.1 ± 1.21	0.749
MOCA score (mean ± SD)	28.31 ± 1.25	27.31 ± 2.28	28 ± 1.06	0.294
RBDSQ (mean ± SD)	5.06 ± 3.04	3.66 ± 2.54	3 ± 2.03	0.130

*H & Y stage, Hoen and Yahr stage; ESS, Epworth Sleepiness Scale; UPDRS III, Unified Parkinson's Disease Rating Scale part III; GDS, Geriatric Depression Scale; MoCA, Montreal Cognitive Assessment; RBD, REM Behavior Disorder Screening Questionnaire*.

* *P-value of one-way ANOVA for age at diagnosis, education years and disease duration, Pearson Chi-square for gender, handedness and H&Y stage and Kruskal–Wallis test for ESS, UPSIT, UPRDS part III, GDS scale, MoCA score, and RBDSQ*.

### Neuropsychiatric Measurement

The neuropsychiatric assessment was performed using the 15-item Geriatric Depression Scale (GDS). For sleep disturbance, RBD and EDS were measured with the REM Sleep Behavior Disorder Screening Questionnaire (RBDSQ), and ESS, respectively.

### Data Acquisition

Data used in the preparation of this paper was obtained from Parkinson's Progression Markers Initiative (PPMI) database (www.ppmi-info.org/data/). This dataset was acquired on a 3 Tesla Siemens scanner, producing 64 DWI (repetition time = 7,748 ms, echo time = 86 ms; voxel size: 2.0 × 2.0 × 2.0 mm^3^; field of view = 224 × 224 mm) at b = 1,000 s/mm^2^ and one b0 image along with a 3D T1-weighted structural scan (repetition time = 8.2 ms, echo time = 3.7 ms; flip angle = 8°, voxel size: 1.0 × 1.0 × 1.0 mm^3^; field of view = 240 mm, acquisition matrix = 240 × 240).

### Diffusion MRI Data Processing

Diffusion MRI data were corrected for subject motion, eddy current distortions, and susceptibility artifacts due to the magnetic field inhomogeneity using ExploreDTI toolbox (20).

### Connectometry Analysis

Diffusion data were reconstructed in the MNI space using q-space diffeomorphic reconstruction to obtain the spin distribution function (SDF). A diffusion sampling length ratio of 1.25 was used, and the output resolution was 1 mm.

Diffusion MRI connectometry (19) was performed to compare group differences in a total of 57 subjects. The group difference was quantified utilizing groupwise *t*-test. The SDF was normalized. A t threshold of 2.5 was assigned to select local connectomes, and the local connectomes were tracked using a deterministic fiber tracking algorithm. All tracks generated from bootstrap resampling were included. A length threshold of 40 mm was used to select tracks. The seeding density was 50 seeds per mm^3^. To estimate the false discovery rate, a total of 1,000 randomized permutations were applied to the group label to obtain the null distribution of the track length. The analysis was conducted using DSI Studio (http://dsi-studio.labsolver.org).

### Statistical Analysis

All statistical analyses were performed in the R statistical package (v3.4.3). To compare between group differences, normality of data was assessed by Shapiro–Wilk test. In case of normal distribution and meeting parametric assumptions, demographic and tests were compared using independent sample *t*-test, two-way ANOVA and chi-square tests, unless Kruskal–Wallis and fisher exact tests were used. *P* values < 0.05 were considered as significant.

## Results

### Demographic Data

In total, 16 PD-EDS (56.2% female) and 45 PD-nEDS propensity score matched subjects (64.4% female), were included in the study. As shown in Table 1, except for UPSIT and ESS score, no significant difference was found between any of demographic variables and motor or neuropsychiatric tests between groups. Mean Age of PD-EDS patients was 62.3 ± 8.8 years, they all had disease duration less than a year and average disease duration was 8.2 ± 7.0 months and H&Y score of 1 or 2. PD-nEDS patients had a mean age of 58.4 ± 9.3 years, with a mean disease duration of 7.9 ± 8.3 months and H&Y score of 1 or 2.

### Connectometry Results

#### PD-EDS Patients vs. PD-nEDS Patients

**Figure 1:**
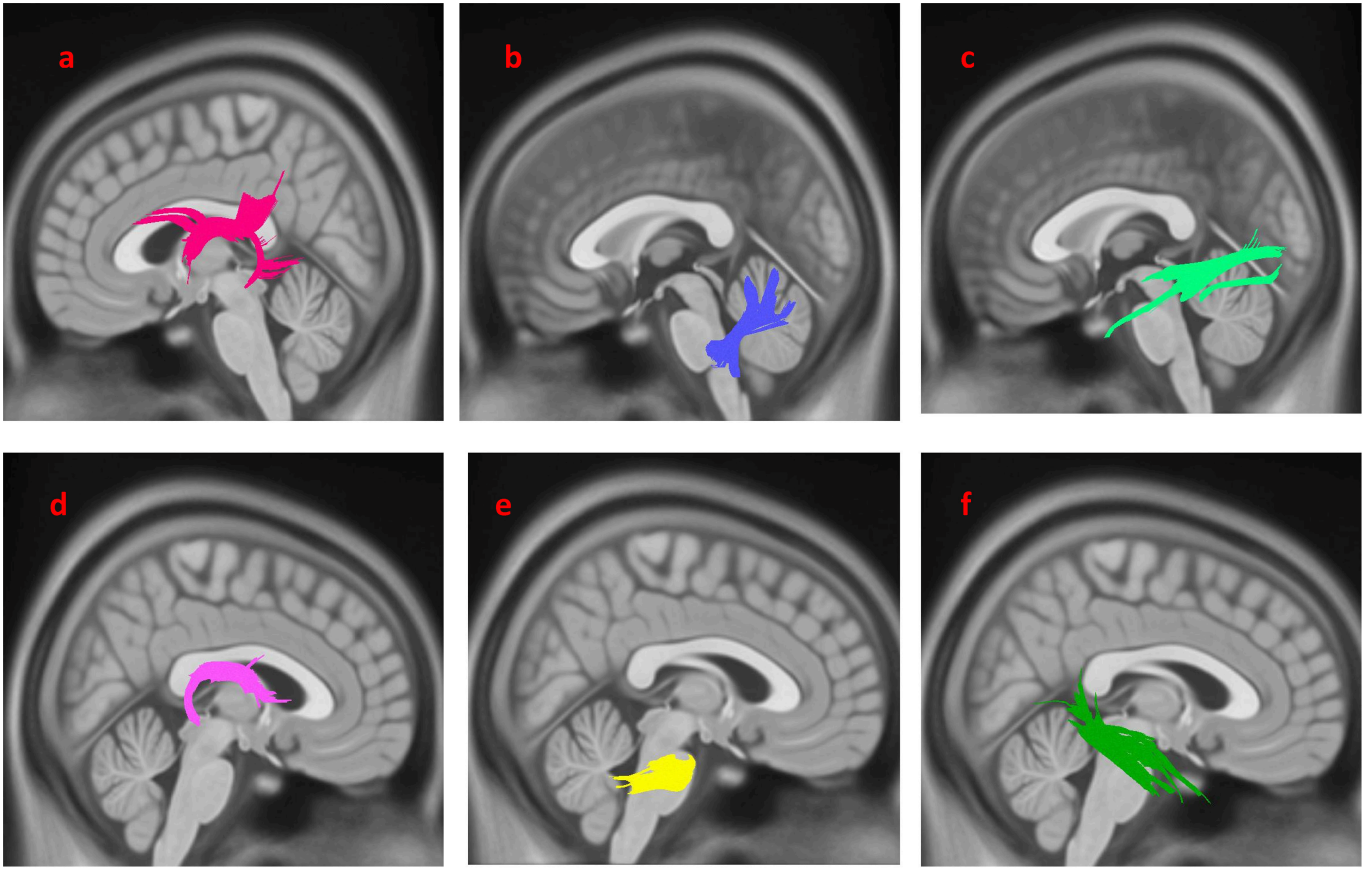
White matter pathways with significantly reduced anisotropy in PD-EDS patients compared to patients without EDS (FDR = 0.021978). **(a)** Left fornix, **(b)** left inferior cerebellar peduncle, **(c)** left inferior longitudinal fasciculus, **(d)** right fornix, **(e)** bilateral middle cerebellar peduncle, **(f)** right inferior longitudinal fasciculus. The results are overlaid on ICBM152 (mni_icbm152_t1) from the McConnell Brain Imaging Center using DSI-STUDIO software.

Compared with PD-nEDS patients, PD-EDS patients showed decreased connectivity in left and right fornix, left, and right inferior longitudinal fasciculus (ILF), middle cerebellar peduncle and left inferior cerebellar peduncle (FDR = 0.021978) (Figure 1).

#### PD-nEDS Patients vs. HC

**Figure 2:**
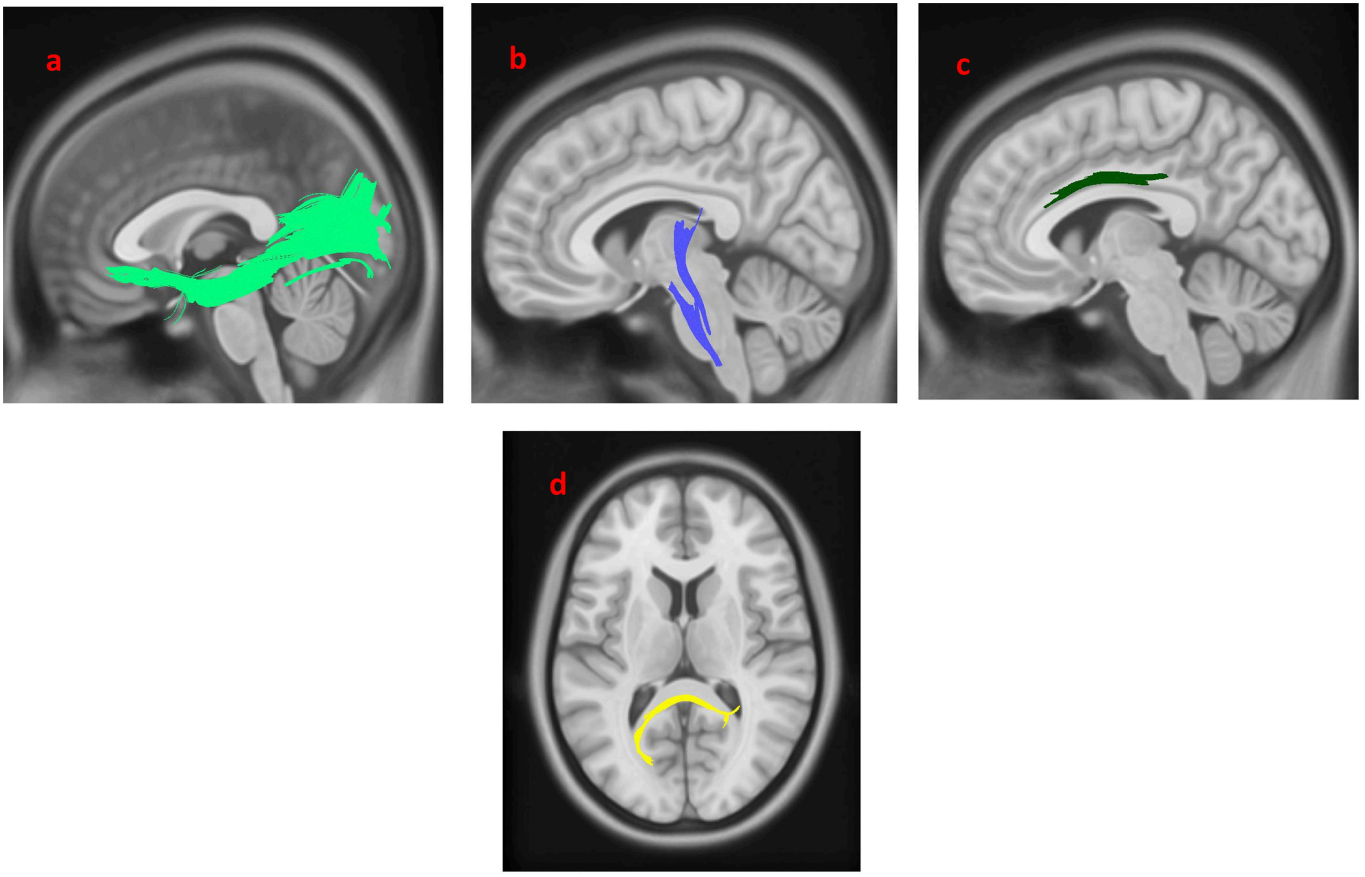
White matter pathways with significantly reduced anisotropy in PD-nEDS patients compared to healthy control (FDR = 0.0277136). **(a)** Left inferior fronto-occipital fasciculus, **(b)** left corticospinal tract, **(c)** left cingulum, **(d)** splenium. The results are overlaid on ICBM152 (mni_icbm152_t1) from the McConnell Brain Imaging Center using DSI-STUDIO software.

As shown in Figure 2, the PD-nEDS group demonstrated decreased connectivity in the left inferior fronto-occipital fasciculus, the splenium, the left corticospinal tract (CST) and the left cingulum contrast to HC (FDR = 0.0277136).

#### PD-EDS Patients vs. HC

**Figure 3:**
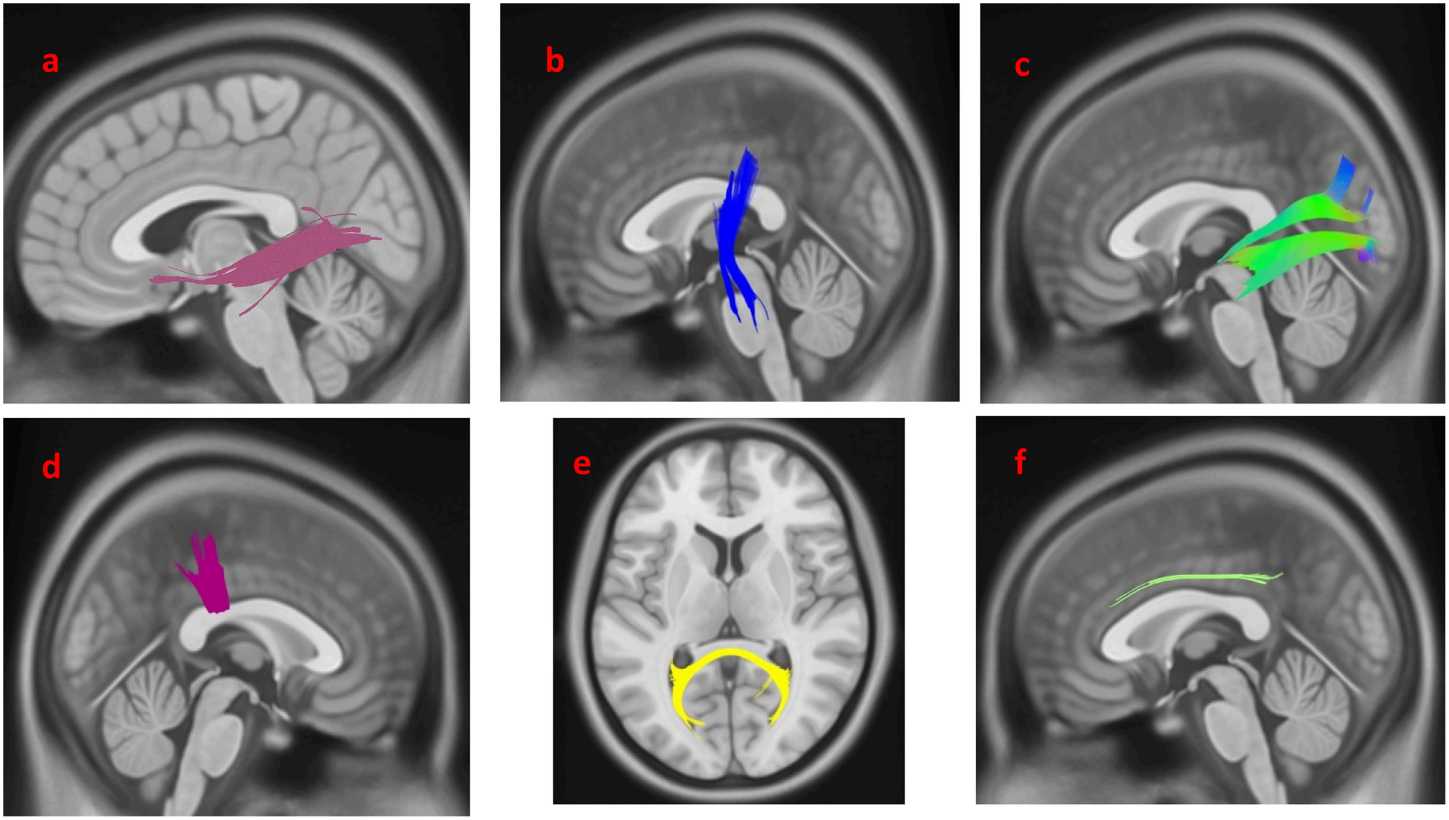
White matter pathways with significantly reduced anisotropy in PD-EDS patients compared to healthy control (FDR = 0.0383761). **(a)** Left inferior fronto-occipital fasciculus, **(b)** left corticospinal tract, **(c)** left inferior longitudinal fasciculus, **(d)** body of the corpus callosum, **(e)** splenium, **(f)** left cingulum. The results are overlaid on ICBM152 (mni_icbm152_t1) from the McConnell Brain Imaging Centre using DSI-STUDIO software.

The group differences between PD-EDS patients and HC are shown in Figure 3. The differences were that connectivity in HC was higher than that in PD-EDS patients in the left inferior fronto-occipital fasciculus, the left ILF, the body of the corpus callosum, the splenium, the left CST and the right cingulum (FDR = 0.0383761).

## Discussion

In this study, we have compared brain white matter microstructure alterations in PD-EDS, PD-nEDS patients, and the control group. Using connectometry, the connectivity (regarding the QA) in the left and right fornix, left and right ILF, middle cerebellar peduncle and left inferior cerebellar peduncle decreased in PD-EDS patients more than it did in PD-nEDS patients. These changes might be concerning the cerebral pathogenesis of EDS in PD patients. It was also demonstrated that connectivity in both PD-EDS and PD-nEDS groups is lower than HC in the left inferior fronto-occipital fasciculus, parts of the corpus callosum, the left CST and cingulum, which are suggested to be involved in PD pathogenesis by previous studies (21–24).

Various mechanisms have been proposed for the etiology of sleep disturbances in PD (25, 26). PD is a subtype of synucleinopathy characterized by alpha-synuclein deposit in neurons and its following induction of neuron degeneration. This course can involve any part of brain including sleep regulating circuits (27). As it is proposed, orexin is one of the most critical neurotransmitters participating in the wakefulness physiology. There are several studies indicating orexin neuron loss in PD patients suffering from sleep disorders. Besides, current evidence supports that the orexin secretion could be partially regulated by dopamine and serotonin (28–30). Studies have indicated that D1 agonists stimulate orexin neurons in animal models while D2 and D3 agonists have a sedative action by blocking the arousal system (31). Consistently, it is revealed that while higher extracellular levels of dopamine promote wakefulness (32), a longtime high-dose use of levodopa disrupts circadian sleep pattern in PD patients due to its inhibitory effect via D2 and D3 receptors (25, 33). However, since we have studied PD patients who were drug-naïve, we can propose that EDS and its correlated changes in tracts such as fornix (and with direct interaction with orexin neurons) are not secondary phenomena and could happen independently from PD treatment.

A DTI study in 2006 indicated reduced fractional anisotropy in the fornix in PD-EDS group, being correlated with ESS score as well (15). The results of our study also show decreased connectivity in the left and right fornix in PD-EDS, compared to healthy controls and patients without EDS. As it was mentioned before, the role of fornix could be explained by the tracts passing through it. Fornix is mainly constructed by fibers connecting hypothalamus to hippocampus. Hypothalamus serves as a fountain for orexin secreting neurons and have global connections with many areas. Studies have shown that the disruption of these tracts and also hypothalamic neuron degeneration (that is also associated with a lower level of orexin) could influence the sleep cycle (15, 34, 35).

DTI analysis by Chondrogiorgi et al. indicated that AD values are increased in the SLF in PD patients with EDS, similar to our study suggesting FA reduction in SLF being related to the development of EDS (14). Besides, our analysis showed that ILF connectivity is decreased in patients with EDS. While involvement of SLF was suggested in patients with impaired alertness in patients having ischemic brain injury, ILF involvement was shown in a group of PD patients with RBD and depressive symptoms (36, 37). These results suggest that mentioned tracts might contribute to EDS neuropathogenesis in PD patients.

Using rs-fMRI research, it was shown that EDS is closely related to the thalamocortical connectivity alternation (38). In another rs-fMRI study using PPMI dataset, Wen et al. indicated that although PD patients with EDS have decreased ReHo in the inferior frontal gyrus and left cerebellum, they have increased ReHo in the left paracentral lobule (16). FC analysis regarding these three regions in PD-EDS group revealed decreased FC of the left cerebellum posterior lobe with the right insula (16). In the past, it was believed that the cerebellum is only involved in motor functions (16). However, recent studies have suggested a role for the cerebellum in sleep-related disorders such as EDS (39, 40). In fact, it is suggested that cerebellum is involved in adjusting various nerve functions such as cognition, emotion, and movement in association with the frontal lobe. It has been indicated by DelRosso and Hoque that this association is weaker in individuals with sleep deprivation compared to individuals with normal sleep (39). Results from our study also show decreased connectivity in middle and left inferior cerebellar peduncle in PD patients with EDS.

Moreover, Gama et al. indicated that patients with EDS suffer from more severe atrophy of medial cerebellar peduncle in comparison with patients without EDS in their gray matter imaging work (12). Another study evaluating GM by Kato et al. demonstrated that PD-EDS patients have significant widespread gray matter atrophy compared to PD-nEDS and controls. Involvement of gray matter in PD-EDS patients was similar to PD patients with dementia (13). This result suggested that EDS could be one of the first presentations of dementia in PD. This study also showed that the basal forebrain atrophy occurs in PD patients, especially PD-EDS patients, and in the substantia innominata, which may be related to the cholinergic neurons loss and corresponds to the regional atrophy of cortical gray matter (13). Moreover, a recent study has revealed that the EDS score was lower in controls than both PD without dementia and PD with dementia (41).

Several limitations should be considered interpreting the results of this study. First, there was a limited number of cases with EDS, having diffusion MRI imaging in PPMI database. Second, longitudinal assessment of white matter changes regarding EDS would help to interpret the results in a better way. Finally, the recognition of EDS in PD patients seems to be difficult. Although, using a questionnaire such as the ESS is relatively simple, the use of ESS has been shown to be subjective (42). Using neuroimaging data for recognition of EDS in PD might lead to earlier and more accurate diagnosis, improving patients' quality of life and guiding clinicians to reach better therapeutic options.

In summary, in the early stages of PD, patients with EDS have decreased connectivity in certain tracts such as fornix and cerebellar peduncles compared to patients without EDS and HC group. However, as there has been limited knowledge about the pathophysiology of EDS in PD, future studies using longitudinal approach, are required to clarify the association between EDS and brain structure alternation in PD and might help to explain some of the contradictory findings.

## Ethics Statement

All procedures performed here, including human participants were in accordance with the ethical standards of the institutional research committee and with the 1964 Helsinki declaration and its later amendments or comparable ethical standards. This study was approved by the Ethical Committee of Zahedan University of Medical Sciences (license # Ir.zaums.rec.1397.314).

## Informed Consent

Written Informed consent was obtained from all individual participants included in the study.

## Author Contributions

AA-G, MS, and MA contributed to the conception and design of the study. BM, MMZ, GK, MM, PS, and MA contributed to data collection and analysis. AA-G, MM, GK, and MA contributed to writing and revising the manuscript.

### Conflict of Interest Statement

The authors declare that the research was conducted in the absence of any commercial or financial relationships that could be construed as a potential conflict of interest.
